# Liraglutide Increases VEGF Expression *via* CNPY2-PERK Pathway Induced by Hypoxia/Reoxygenation Injury

**DOI:** 10.3389/fphar.2019.00789

**Published:** 2019-07-24

**Authors:** Chong Liu, Yong Liu, Jing He, Rong Mu, Yanbo Di, Na Shen, Xuan Liu, Xiao Gao, Jinhui Wang, Tie Chen, Tao Fang, Huanming Li, Fengshi Tian

**Affiliations:** ^1^Department of Anaesthesiology, Tianjin 4th Centre Hospital, The Fourth Central Hospital Affiliated to Nankai University, The Fourth Center Clinical College of Tianjin Medical University, Tianjin, China; ^2^Central Laboratory, Tianjin 4th Centre Hospital, The Fourth Central Hospital Affiliated to Nankai University, The Fourth Center Clinical College of Tianjin Medical University, Tianjin, China; ^3^Department of Cardiology, Tianjin 4th Centre Hospital, The Fourth Central Hospital Affiliated to Nankai University, The Fourth Center Clinical College of Tianjin Medical University, Tianjin, China

**Keywords:** liraglutide, angiogenesis, CNPY2, unfolded protein response, hypoxia/reoxygenation

## Abstract

Liraglutide (Lir) is a glucagon-like peptide-1 receptor agonist that lowers blood sugar and reduces myocardial infarct size by improving endothelial cell function. However, its mechanism has not yet been clarified. Unfolded protein response (UPR) plays an important role in the pathogenesis of myocardial ischemia-reperfusion injury. It determines the survival of cells. Endoplasmic reticulum position protein homologue 2 (CNPY2) is a novel initiator of UPR that also participates in angiogenesis. To this extent, the current study further explored whether Lir regulates angiogenesis through CNPY2. In our article, a hypoxia/reoxygenation (H/R) injury model of human umbilical vein endothelial cells (HUVECs) was established and the effect of Lir on HUVECs was first evaluated by the Cell Counting Kit-8. Endothelial tube formation was used to analyze the ability of Lir to induce angiogenesis. Subsequently, the effect of Lir on the concentrations of hypoxia-inducible factor 1α (HIF1α), vascular endothelial growth factor (VEGF), and CNPY2 was detected by enzyme-linked immunosorbent assay. To assess whether Lir regulates angiogenesis through the CNPY2-initiated UPR pathway, the expression of UPR-related pathway proteins (CNPY2, GRP78, PERK, and ATF4) and angiogenic proteins (HIF1α and VEGF) was detected by reverse transcription-polymerase chain reaction and Western blot. The results confirmed that Lir significantly increased the expression of HIF1α and VEGF as well as the expression of CNPY2-PERK pathway proteins in HUVECs after H/R injury. To further validate the experimental results, we introduced the PERK inhibitor GSK2606414. GSK2606414 was able to significantly decrease both the mRNA and protein expression of ATF4, HIF1α, and VEGF in vascular endothelial cells after H/R injury. The effect of Lir was also inhibited using GSK2606414. Therefore, our study suggested that the CNPY2-PERK pathway was involved in the mechanism of VEGF expression after H/R injury in HUVECs. Lir increased the expression of VEGF through the CNPY2-PERK pathway, which may promote endothelial cell angiogenesis and protect HUVEC from H/R damage.

## Introduction

Acute myocardial infarction (AMI) is a type of cardiovascular disease that occurs after acute coronary artery occlusion, which results in myocardial hypoxia and necrosis. Known as a silent killer, the incidence and mortality rate of AMI worldwide are on the rise and the prognosis is extremely poor. After AMI, it is crucial to restore coronary blood supply as soon as possible to mitigate myocardial ischemic injury (Santos-Gallego et al., 2014; Prondzinsky et al., 2018). Reperfusion methods such as percutaneous coronary intervention, thrombolysis, and surgical bypass have successfully restored coronary blood supply and limited the infract size of the myocardium (Boateng and Sanborn, 2013). However, a portion of the cardiac tissue still does not perfuse properly due to damage to the microvasculature, that is, no reflow (Kloner et al., 1974). The coronary no-reflow phenomenon is an independent predictor of persistent myocardial ischemia, ventricular remodeling, and dysfunction after coronary reperfusion (Bouleti et al., 2015; Mazhar et al., 2016; Rezkalla et al., 2017), as it affects immediate and long-term prognosis (Ndrepepa et al., 2010; Choo et al., 2014). Therefore, the current treatment methods cannot fundamentally solve the problem of myocardial injury, forcing us to explore a new treatment or an adjuvant treatment.

The angiogenesis or reopening of residual blood vessels in the infarct area is the key to affect the survival and functional status of cardiomyocytes (Lutter et al., 2004). In recent years, promoting angiogenesis has become an attractive and novel strategy for the treatment of acute infarction. In the process of angiogenesis, endothelial cells are important target cells and participate in the entire process (Gaengel et al., 2009; Arima et al., 2011; Liu et al., 2018b). Recent studies have shown that unfolded protein response (UPR) is involved in angiogenesis (Paridaens et al., 2014; Binet and Sapieha, 2015). When myocardial cells were stimulated by ischemia and hypoxia, intracellular calcium overload and unfolded proteins in the lumen of the endoplasmic reticulum (ER) increased significantly, inducing UPR (Groenendyk et al., 2013; Zhang et al., 2017). UPR is a complex signaling pathway that determines cell survival (Shaheen, 2018). When cells are exposed to one or more angiogenesis stimuli (e.g., hypoxia/ischemia, inflammation, and oxidative stress), a large number of misfolded, unfolded proteins accumulate in the ER and calcium imbalance occurs (Binet and Sapieha, 2015). The UPR is activated and involved in the survival and activity of endothelial cells (Luchetti et al., 2017). However, the mechanism behind UPR sensing is not fully understood (Ron and Walter, 2007; Wolff et al., 2014). Recent studies have found that Canopy 2 (canopy fiber growth factor signaling regulator 2, CNPY2) participates in UPR in the ER, which is the main trigger of the PERK-CHOP pathway (Hong et al., 2017). CNPY2 belongs to the Canopy family (CNPY1–CNPY4) and is widely found in the nervous, cardiovascular, respiratory, digestive, and reproductive systems (Hatta et al., 2014; Guo et al., 2015b). During transition of ER from nonstressed to stressed state, the CNPY2 binding partner switches from GRP78 to PERK and selectively initiates the PERK-CHOP-mediated apoptotic signaling pathway (Urra and Hetz, 2017). Some studies have found that CNPY2 can enhance angiogenesis and promote smooth muscle cell migration and proliferation (Guo et al., 2015a; Guo et al., 2015b). Therefore, the UPR plays an important role in the angiogenesis process after AMI and has gradually attracted widespread attention.

Liraglutide (Lir) is a glucagon-like peptide-1 (GLP-1) analogue that binds the same endogenous receptor to stimulate insulin secretion (Greig and Scott, 2015). In recent years, Lir has been found to have other pharmacological benefits, including the reduction of inflammatory response and myocardial infract size, which improved overall cardiovascular function (Dai et al., 2013; Chen et al., 2016; Hu et al., 2017). Clinical studies have shown that, in addition to its ability to control hyperglycemia, Lir was able to reduce weight and blood pressure, which is beneficial to patients with type 2 diabetes and cardiovascular diseases (Marso et al., 2016; Guthrie, 2018). Previous studies have demonstrated that GLP-1 induced angiogenesis in human endothelial cells *via* the protein kinase B, Src, and protein kinase C pathways *in vitro* (Aronis et al., 2013). Experimental studies revealed that Lir can down-regulate high-glucose-induced UPR activation and ER stress (ERS) in cultured endothelial cells (Schisano et al., 2012). More studies have found that Lir reduced endothelial cell ERS and insulin resistance in diabetic patients (Breton-Romero et al., 2018). In addition, studies have shown that Lir improved the no-reflow rate after MI (Chen et al., 2016), suggesting that it plays a key role in reducing myocardial ischemia-reperfusion injury (MIRI). However, the mechanism behind its function in the ischemic heart is still unclear.

Therefore, based on UPR-mediated angiogenesis and the cardioprotective effect of Lir, the purpose of this study is to further provide a theoretical basis for the treatment of angiogenesis after MI.

## Materials and Methods

### Main Reagents

Lir, also known as Victoza, was purchased from Novo Nordisk A/S (Copenhagen, Denmark). GSK2606414, a PERK inhibitor, was purchased from the Merck KGaA Group (Darmstadt, Germany). Dimethyl sulfoxide (DMSO) was purchased from Sigma-Aldrich (St. Louis, MO, USA). The Cell Counting Kit-8 (CCK-8) kit was purchased from Beijing Solarbio Science & Technology Co., Ltd. (Beijing, China). The human CNPY2 enzyme-linked immunosorbent assay (ELISA) kit was purchased from Proteintech Group (Chicago, IL, USA). The hypoxia-inducible factor 1α (HIF1α) and vascular endothelial growth factor (VEGF) ELISA kits were purchased from Abcam (Cambridge, UK).

To increase the physiology-related possibilities observed in these studies, human umbilical vein endothelial cells (HUVECs) were used in the experiment. In addition, the concentration of Lir used was 100 nM, which is within the therapeutic range of 1.8 mg/day human injection of Victoza, a widely used clinical brand of Lir (Krasner et al., 2014).

### HUVECs Culture

HUVECs were purchased from the National Experimental Cell Resource Sharing Platform and cultured in Dulbecco’s modified Eagle medium (DMEM; Gibco, USA) containing 10% fetal bovine serum (FBS; ausgenex Australia). The cultured HUVECs were incubated in a saturated humidity chamber containing 95% air and 5% CO_2_ at 37°C. When cell confluence reached about 90%, the cells were subcultured. Cells in passages 4 to 6 with healthy architecture were selected for subsequent experiments.

### Establishing the Hypoxia/Reoxygenation (H/R) Model of HUVECs

When cells were in logarithmic growth phase, the H/R injury model was established as described above (Gao et al., 2017). The original medium was replaced by hypoxic medium without FBS and glucose, and the cells were cultured in a hypoxic chamber of 5% CO_2_ and 95% N_2_ for 1.5 h at 37°C. Then, the cell culture medium was replaced back into DMEM containing 10% FBS and reoxygenated for 24 h under normal oxygen content.

### Grouping and Treatment of HUVECs

The cultured cells were randomly divided into four groups: (1) Sham group, without H/R; (2) Lir group, without H/R, only Lir treatment (100 nM); (3) H/R group, hypoxic treatment for 1.5 h followed by reoxygenation for 24 h; and H/R+Lir group, with Lir treatment (100 nM) after 1.5 h hypoxia treatment and then reoxygenation for 24 h. Sham group and H/R group were given an equal volume of DMEM as Lir. All experiments were repeated three times.

To further elucidate the potential mechanism of the CNPY2-PERK pathway-mediated angiogenesis, we introduced a PERK inhibitor GSK2606414 (G, 0.03 μM, soluble in DMSO; Axten et al., 2012). Next, the cultured cells were randomly divided into eight groups: 1) Sham group, without H/R; 2) Lir group, without H/R, only added Lir (100 nM); 3) H/R group, treated with hypoxia for 1.5 h followed by reoxygenation for 24 h; 4) H/R+Lir group, treated with Lir (100 nM) after 1.5 h hypoxia treatment followed by reoxygenation for 24 h; 5) GSK2606414 group (G group), without H/R, only added GSK2606414 (0.03 μM); 6) G+Lir group, cells were treated with GSK2606414 (0.03 μm) and Lir (100 nM) without H/R followed by 24 h reoxygenation; 7) H/R+G group, cells were treated with GSK2606414 (0.03 μM) after 1.5 h hypoxia treatment followed by 24 h reoxygenation; and 8) H/R+G+Lir group, cells were treated with GSK2606414 (0.03 μM) and Lir (100 nM) after 1.5 h hypoxia treatment followed by 24 h reoxygenation. Sham group, H/R group, G group, and H/R+G group were given an equal volume of DMEM with Lir. Sham group, Lir group, H/R group, and H/R+Lir group were added to an equal volume of DMSO with GSK2606414.

### Cell Viability Assay

Cell viability was determined by CCK-8 as described previously (Tu et al., 2017). Briefly, HUVECs were inoculated into 96-well plates at a concentration of 5×10^3^ cells/well in 100-μl medium. After 24 h cell culture, 100 nM Lir was added to the cells in groups, and then 10 μl CCK-8 solution was added at 6, 12, 18, 24, 30, and 36 h reoxygenation, respectively. Cells were incubated in an incubator at 37°C for 2 h. The absorbance was measured at 450 nm using a Swiss TECAN Infinite F50 microplate reader. Cells cultured in serum-free DMEM were used as a background control. All experiments were performed three times.

### Tube Formation Assay

Endothelial cell tube formation assay is a useful indicator of angiogenesis potential. Matrigel angiogenesis assay was performed as described previously (Cheng et al., 2019). Briefly, HUVECs (5×10^4^/well) were seeded onto 24-well plates precoated with Matrigel (200 μl). The cells were randomly divided into four groups: H/R group, H/R+Lir group, H/R+G group, and H/R+G+Lir group. After 1.5 h hypoxia, 100 nM Lir was added to cells in Lir-positive groups and cultured at 37°C for 24 h. Three sites in each well were randomly selected after 24 h reoxygenation and photographed using an inverted microscope (100× magnification). The number of nodes and the total tube length were calculated by ImageJ software to analyze the tube formation.

### Enzyme-Linked Immunosorbent Assay (ELISA)

Concentrations of CNPY2, HIF1α, and VEGF were determined by individual ELISA kits according to the manufacturer’s instructions. Briefly, the culture medium of each group was centrifuged at 3,000×g for 5 min, and 300 μl supernatant was used for the ELISA kit. Phosphate-buffered saline was used as a control. The absorbance was measured at 450 nm.

### RNA Extraction and Reverse Transcription-Polymerase Chain Reaction Analysis

According to the manufacturer’s instructions, total RNA was isolated from HUVECs using RNeasy Mini Kit (Qiagen, Germany). cDNA was synthesized using the PrimeScript^™^ reverse transcription-polymerase chain reaction (RT-PCR) Kit (TAKARA, Japan). The amplification conditions of PCR were as follows for 35 cycles: predenaturation at 95°C for 5 min, 95°C for 30 s, 60°C for 30 s, 72°C for 40 s, and elongation at 72°C for 7 min. All reactions were performed three times. Primers were synthesized using Sangon Biotech Co. Ltd. Primers sequences used were as follows: CNPY2 (forward 5′-ATTGATCCTTCCACCCATCGC-3′ and reverse 5′-CAATGCTCTCACACGCAAACT-3′), GRP78 forward 5′-CCAAGAACCAGCTCACCTCC-3′ and reverse 5′-ACCACCTTGAACGGCAAGAA-3′, HIF1α (forward 5′-GGCAGCAACGACACAGAAAC-3′ and reverse 5′-GCGTTTCAGCGGTGGGTAAT-3′), VEGF (forward 5′-ATTGGAGCCTTGCCTTGCT-3′ and reverse 5′-AGCTGCGCTGATAGACATCC-3′), calnexin (forward 5′-AGATGACTGGGATGAAGATGCC-3′ and reverse 5′-CACAGCTCCAAACCAATAGCAC-3′), and β-actin (forward 5′-GGTCAGAAGGATTCCTATGTG-3′ and reverse 5′-ATTGCCAATGGTGATGACCTG-3′). Calnexin or β-actin was used as a reference gene. mRNA data were expressed by the ratio of the target gene to the reference gene. Relative expression was calculated according to the intensity of the bands using the Image Lab software (Bio-Rad, USA).

### Protein Preparation and Western Blot

HUVECs were lysed with ice-cold radioimmunoprecipitation assay buffer (Solarbio) and the concentration was measured by Bicinchoninic Acid Assay Kit (Sigma, USA). The cell lysate was centrifuged at 12,000×g for 30 min at 4°C, and the supernatant was the total cell lysate. To isolate the ER from HUVECs, the cell lysate was centrifuged (4°C, 800×g) for 10 min, and the new supernatant was centrifuged (4°C, 10,000×g) for 20 min; the new supernatant was again collected for centrifugation (4°C, 100,000×g) for 60 min. The final precipitate is ER, which is suspended in a pyrolysis solution containing 1% Triton X-100 (Sigma; Qi et al., 2007). Protein samples (30 μg/lane) were subject to 10% sodium dodecyl sulfate-polyacrylamide gel electrophoresis and transferred onto polyvinylidene fluoride membrane (Millipore, Billerica, MA). Membranes were blocked with 5% skim milk at 4°C for 1 h and incubated with the primary antibodies overnight. The primary antibodies were as follows: CNPY2 (1:1,000; Proteintech Group), GRP78 (1:2,000; Abcam), PERK (1:1,000; Cell Signaling Technology, USA), p-PERK (1:1,000; Cell Signaling Technology), ATF4 (1:1:5,000; Abcam), HIF1α (1:1,000; Abcam), VEGF (1:1,000; Abcam). Calnexin (1:250; Abcam) and β-actin (1:5,000; Abcam) served as internal controls. After three washes, the membrane was incubated with the corresponding horseradish peroxidase-conjugated secondary antibody (1:10,000; Solarbio) for 1 h at room temperature. Target bands were visualized using enhanced chemiluminescence (Merck Millipore, Billerica, MA, USA) methods. The quantification of the blot was measured using Image Lab software. All band intensities were normalized to calnexin or β-actin and expressed as a percentage of the control sample.

### Statistical Analysis

All experiments in this study were performed at least three times. Data are expressed as mean ± SD. Comparisons among multiple groups were performed by one-way analysis of variance followed by the Student’s-Newman-Keuls’ *post hoc* test. All statistical analyses were performed using GraphPad Prism 5 software (GraphPad Software, San Diego, CA, USA). P < 0.05 is considered to be statistically significant

## Results

### Lir Enhanced the Activity and Tube Formation After H/R Injury in HUVECs

**Figure 1:**
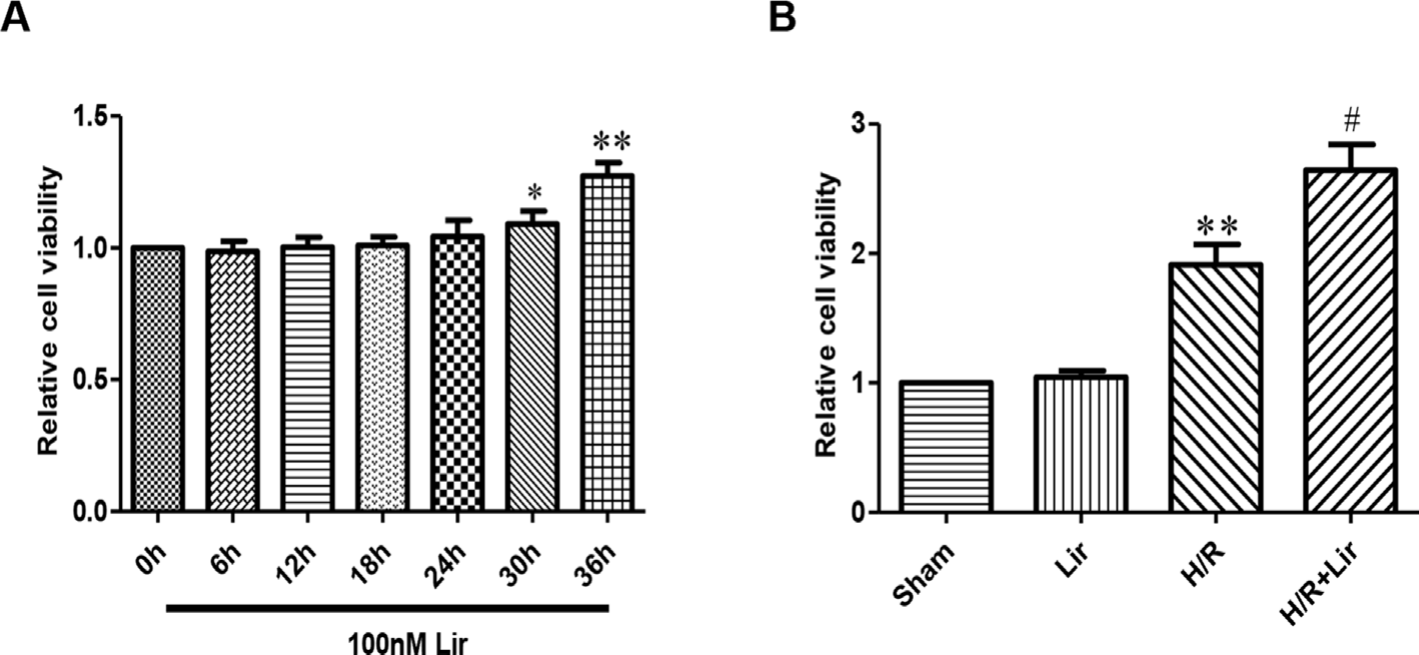
Effect of liraglutide (Lir) on human umbilical vein endothelial cell (HUVEC) viability. **(A)** Effect of Lir on the viability of untreated HUVECs at different time points. **(B)** Effect of Lir on the viability of HUVECs after H/R injury. Data are mean ± SE. *P < 0.05, **P < 0.01 compared to Sham group; ^#^P < 0.05, ^##^P < 0.01 compared to H/R group.

The effect of Lir at 100 nM on HUVECs viability was examined at different time points (0, 6, 12, 18, 24, 30, and 36 h). Under the same conditions, cell viability rate increased significantly after Lir treatment for more than 24 h (P < 0.05; **Figure 1A**). The results suggested that 24 h is the cell logarithmic growth phase promoted by Lir. Therefore, we chose a treatment time of 24 h for subsequent studies.

**Figure 2:**
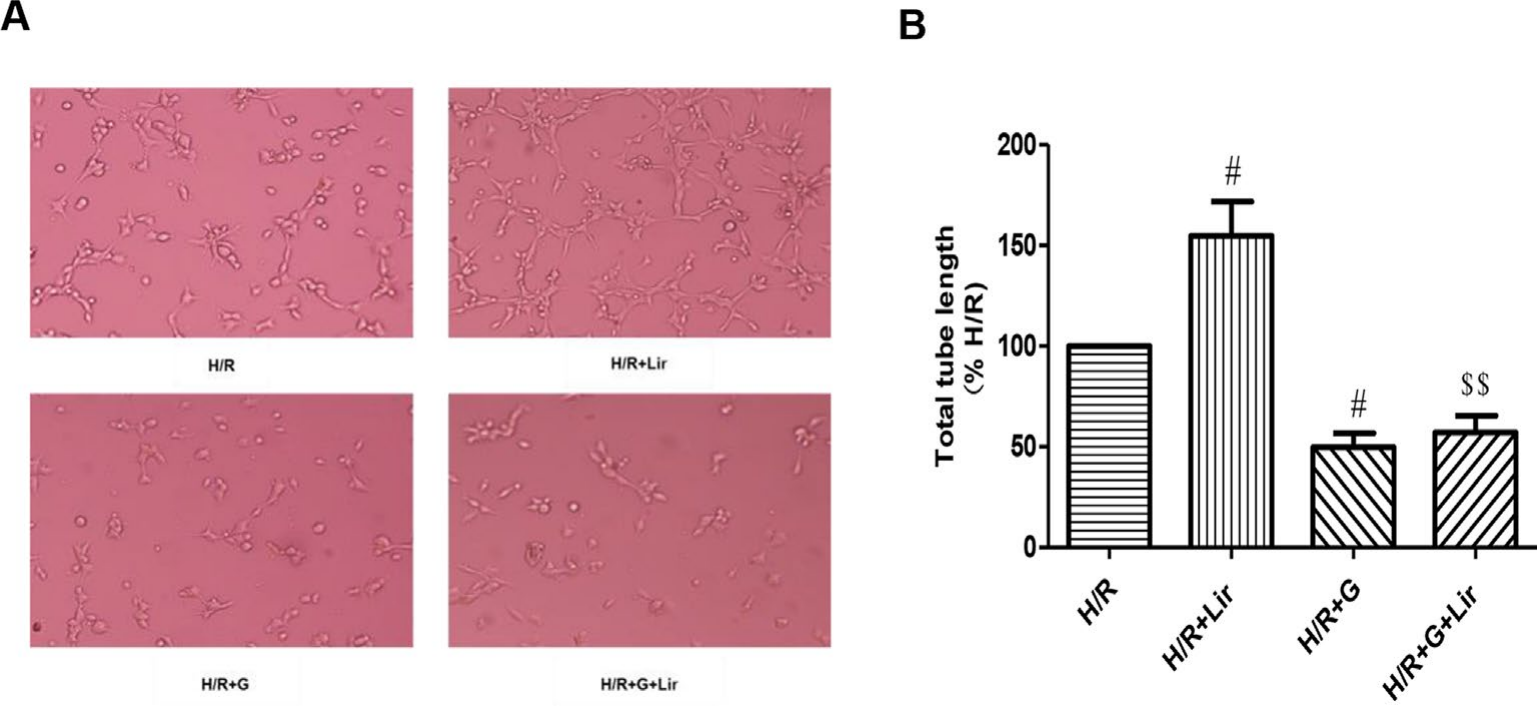
Effect of Lir on the tube formation after H/R injury in HUVECs at different time points. Bar graph of tube formation assessment with different times of Lir. Data were analyzed as tube percentage versus control group. ^#^P < 0.05, ^##^P < 0.01 compared to H/R group; ^$^P < 0.05, ^$$^P < 0.01 compared to H/R+Lir group.

Subsequently, to determine the effect of Lir on HUVEC viability after H/R injury, we used the CCK-8 assay. There was no significant effect on cell viability in Sham group and Lir group. Compared to Sham group, cell viability in H/R group was significantly increased. The application of Lir further enhanced cell viability, which was significantly higher than in H/R group (P < 0.01; **Figure 1B**). Endothelial cell tube formation assay is a useful indicator of angiogenic potential. To further validate the effect of Lir on angiogenesis, we examined changes of cell tube formation after H/R injury in HUVECs. The experimental results showed that cell tube formation in H/R+Lir group was significantly higher than that in H/R group 24 h after reoxygenation, and the PERK inhibitor significantly reduced this effect (P < 0.01; **Figure 2**). The results suggested that Lir enhanced the activity and tube formation after H/R injury in HUVECs.

### Lir Increased the Expression of HIF1α and VEGF After H/R Injury in HUVECs

**Figure 3:**
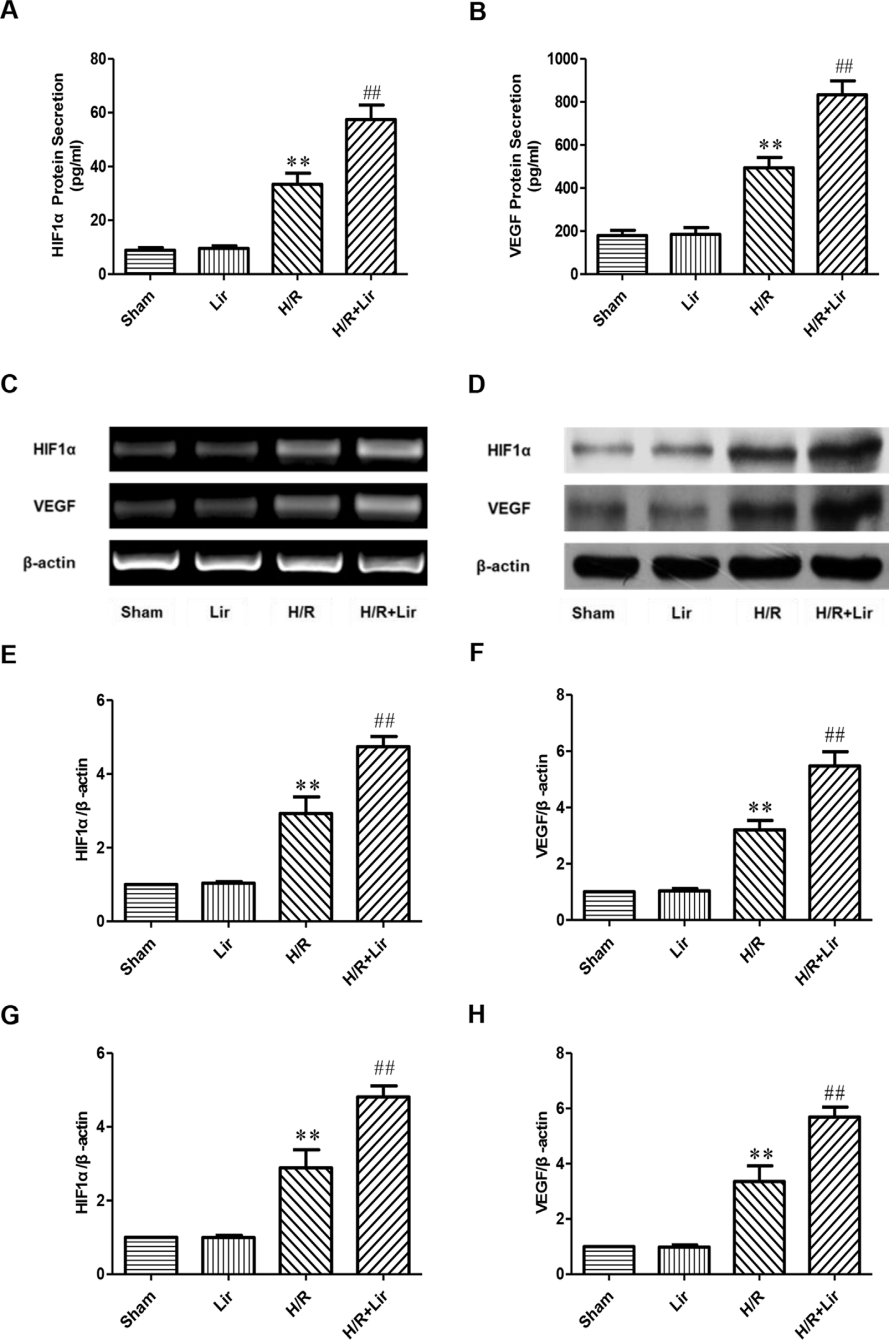
Effect of Lir on HIF1α and VEGF after H/R injury in HUVECs. **(A)** Effect of Lir on the protein secretion of HIF1α after H/R injury. **(B)** Effect of Lir on the protein secretion of VEGF after H/R injury. **(C)** Effect of Lir on the mRNA expression level of HIF1α and VEGF after H/R injury. **(D)** Effect of Lir on the protein expression level of HIF1α and VEGF after H/R injury. **(E)** mRNA expression level of HIF1α. **(F)** mRNA expression level of VEGF. **(G)** Protein expression level of HIF1α. **(H)** Protein expression level of VEGF. Results were normalized to the percentage of β-actin expression. Data are mean ± SE. *P < 0.05, **P < 0.01 compared to Sham group; ^#^P < 0.05, ^##^P < 0.01 compared to H/R group.

Previous studies have confirmed that factors such as HIF1α and VEGF play a key role in angiogenesis. To determine the effect of Lir on angiogenesis after H/R injury, secreted HIF1α and VEGF in each group were analyzed by ELISA kit. After H/R injury, HIF1α was significantly elevated compared to Sham group. Lir treatment further increased HIF1α production compared to H/R (P < 0.01; **Figure 3A**). The protein level of HIF1α in Lir alone group was slightly higher than in Sham group, but it was not statistically significant. In contrast, VEGF level was similar between Sham group and Lir group. After H/R injury, VEGF production was significantly enhanced, whereas the VEGF content in H/R+Lir group was significantly higher than that in H/R group (P < 0.01, **Figure 3B**). Meanwhile, to verify ELISA results, we subsequently detected the mRNA and protein expression of HIF1α and VEGF (**Figure 3C and D**). Compared to Sham group, mRNA expression levels of HIF1α and VEGF were significantly up-regulated after H/R injury. After treatment with Lir, HIF1α and VEGF were further increased compared to H/R group (P < 0.01; **Figure 3E and F**). Consistent with the mRNA results, protein expression of HIF1α and VEGF in H/R group was significantly higher than in Sham group, which was further enhanced after Lir treatment (P < 0.01; **Figure 3G and H**). These results demonstrated that Lir significantly increased the synthesis and expression of HIF1α and VEGF, suggesting that Lir may augment angiogenesis of HUVECs after H/R injury.

### Lir Increased the Expression of CNPY2 After H/R Injury in HUVECs

**Figure 4:**
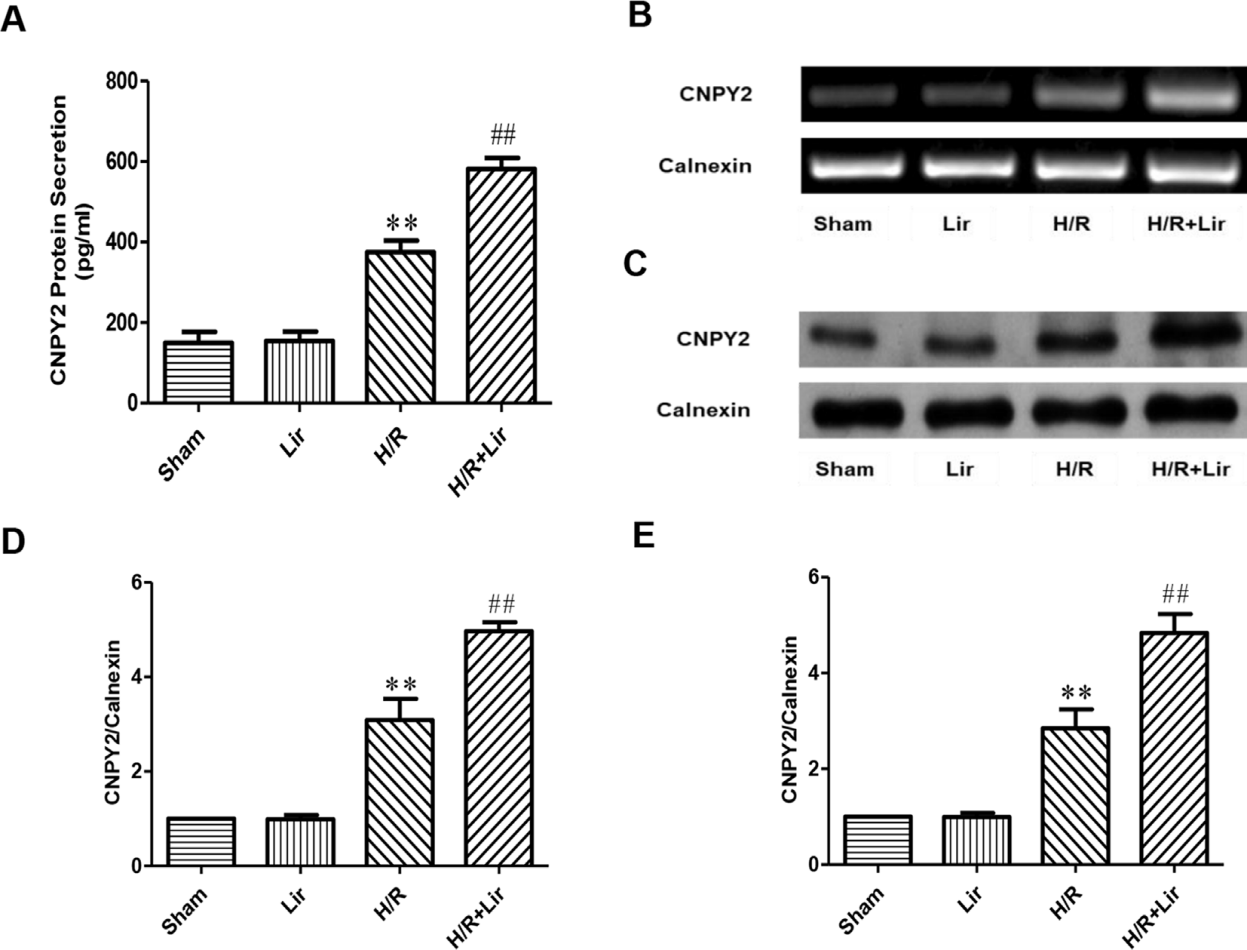
Effect of Lir on CNPY2 after H/R injury in HUVECs. **(A)** Protein secretion of CNPY2. **(B)** mRNA expression level of CNPY2 from each group. **(C)** Protein expression level of CNPY2 from each group. **(D)** mRNA expression level of CNPY2. **(E)** Protein expression level of CNPY2. Results were normalized to the percentage of calnexin expression. Data are mean ± SE. *P < 0.05, **P < 0.01 compared to Sham group; ^#^P < 0.05, ^##^P < 0.01 compared to H/R group.

Studies have shown that the secreted protein CNPY2 is highly expressed in the cardiovascular system, and it has also been established that CNPY2 promotes angiogenesis in smooth muscle cells. To confirm the high expression of CNPY2 in HUVECs and the effect of Lir treatment on CNPY2, we first determined the concentration of CNPY2 in the culture medium of HUVECs by ELISA. The results showed that the expression of CNPY2 was detected in both Sham group and Lir group, and there was no significant difference between the two groups. However, the concentration of CNPY2 was significantly increased under H/R for 24 h (P < 0.01; **Figure 4A**). Second, we detected the mRNA expression of CNPY2 in HUVECs (**Figure 4B**). After 24 h H/R injury, mRNA of CNPY2 was significantly increased compared to Sham group. After treatment with Lir, CNPY2 mRNA expression was significantly higher than in H/R group (P < 0.01; **Figure 4D**). To further verify the above results, we further examined the protein expression of CNPY2 (**Figure 4C**). Consistent with the results of ELISA and RT-PCR, the protein expression of CNPY2 was significantly increased after Lir treatment compared to H/R group (P < 0.01; **Figure 4E**). These results indicated that CNPY2 was highly expressed in HUVECs, and treatment with Lir further increased the expression of CNPY2 after H/R, an important factor in angiogenesis.

### Lir Promoted Angiogenesis Through the CNPY2-PERK Pathway After H/R Injury in HUVECs

**Figure 5:**
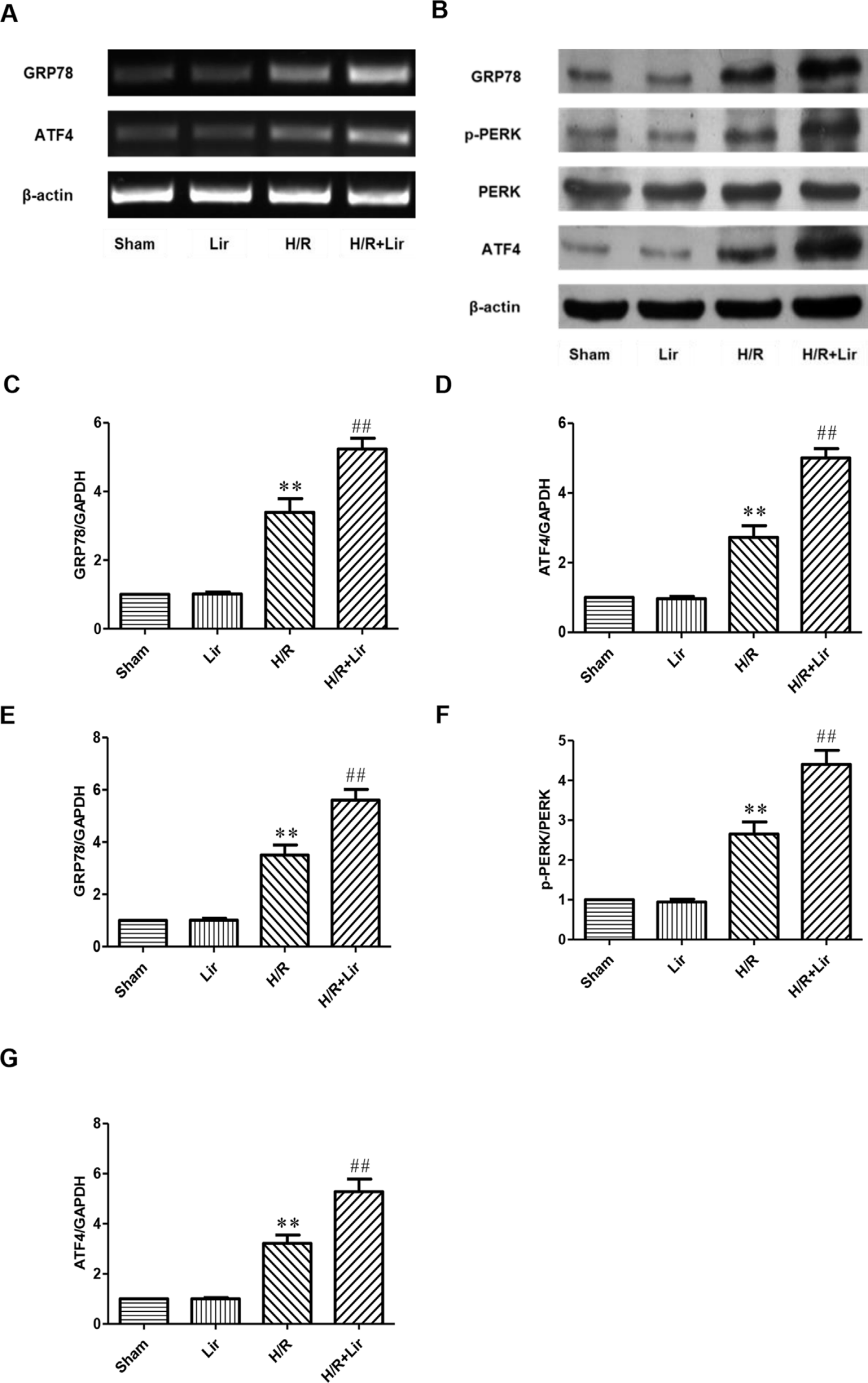
Effect of Lir on the ERS-related pathway induced by H/R injury in HUVECs. **(A)** mRNA expression levels of ERS markers were determined by RT-PCR. **(B)** Protein expression levels of ERS markers were determined by Western blot. mRNA expression levels of ERS marker proteins GRP78 **(C)** and ATF4 **(D)**. Protein expression levels of ERS marker proteins GRP78 **(E)**, p-PERK **(F)**, and ATF4 **(G)**. Expression of β-actin was used for the loading control. Results were normalized to the percentage of β-actin expression. Data are mean ± SE. *P < 0.05, **P < 0.01 compared to Sham group; ^#^P < 0.05, ^##^P < 0.01 compared to H/R group.

To further investigate whether Lir promotes angiogenesis *via* the CNPY2-PERK pathway after H/R, we examined the expression of CNPY2-PERK pathway-related proteins (**Figure 5A and B**). As shown in **Figure 5**, there was no significant difference in the mRNA and protein expression levels of GRP78 and ATF4 between Sham group and Lir group (P > 0.05). After H/R injury, the mRNA and protein expression levels were significantly increased compared to Sham group. The application of Lir promoted the mRNA and protein expression of GRP78 and ATF4 compared to H/R group (**Figure 5C–G**). These results indicated that Lir might promote angiogenesis *via* the CNPY2-PERK pathway.

**Figure 6:**
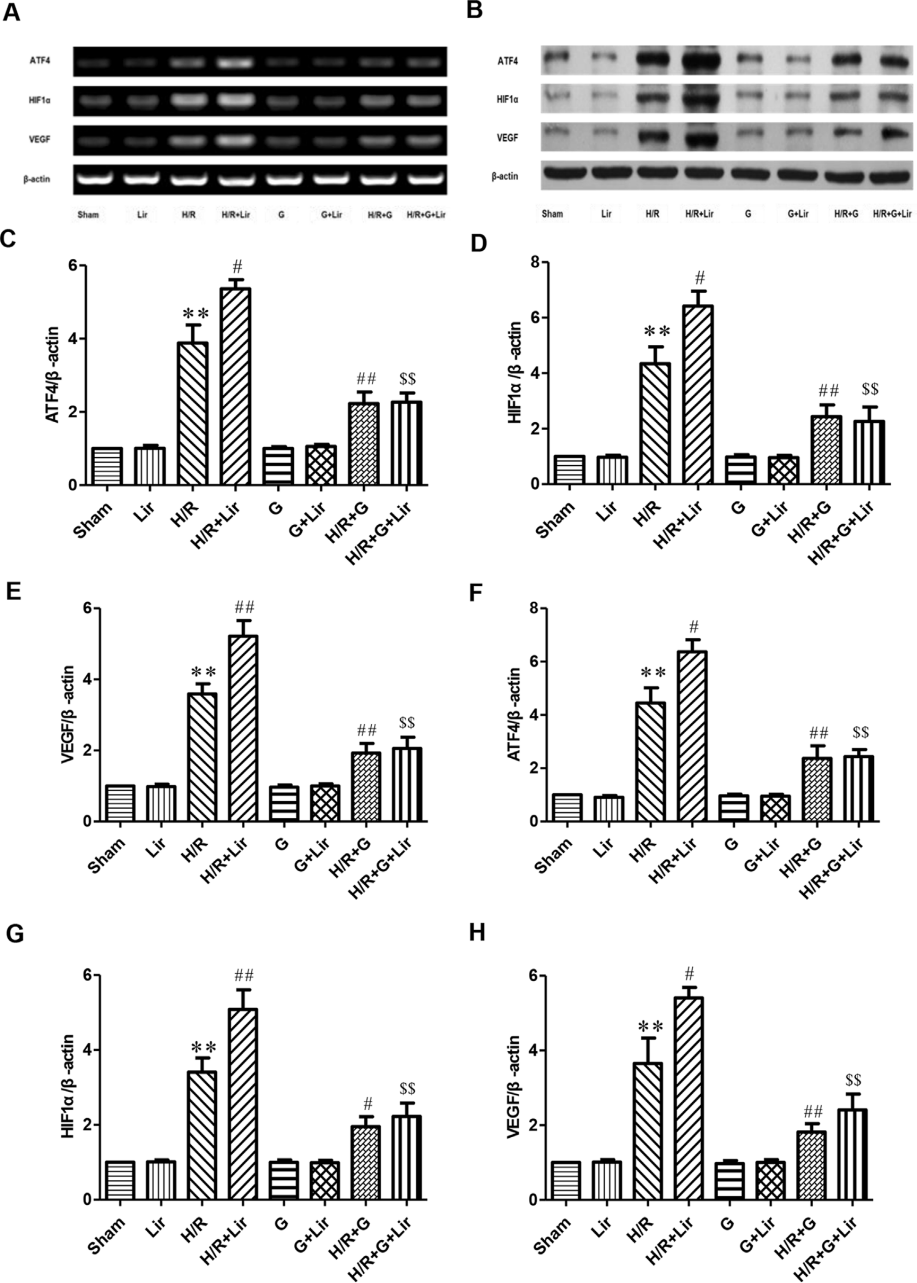
Effect of Lir on the CNPY2-PERK pathway induced by H/R injury in HUVECs. **(A)** mRNA expression levels of the CNPY2-PERK pathway proteins were determined by RT-PCR. **(B)** Protein expression levels of the CNPY2-PERK pathway proteins were determined by Western blot. mRNA expression levels of the CNPY2-PERK pathway proteins ATF4 **(C)**, HIF1α **(D)**, and VEGF **(E)**. Protein expression levels of CNPY2-PERK pathway proteins ATF4 **(F)**, HIF1α **(G)**, and VEGF **(H)**. Expression of β-actin was used for the loading control. Results were normalized to the percentage of β-actin expression. Data are mean ± SE. *P < 0.05, **P < 0.01 compared to Sham group; ^#^P < 0.05, ^##^P < 0.01 compared to H/R group; ^$^P < 0.05, ^$$^P < 0.01 compared to H/R+Lir group.

Furthermore, to further confirm whether the CNPY2-PERK pathway is involved in the angiogenesis of Lir to HUVECs exposed to H/R, we added the PERK inhibitor GSK2606414 (**Figures 6A, B**). RT-PCR and Western blot results showed no significant difference in the expression of ATF4, HIF1α, and VEGF among Sham group, Lir group, G group, and G+Lir group (P > 0.05; **Figure 6C–H**). The mRNA and protein expression levels were significantly increased in H/R group compared to Sham group (P < 0.01; **Figures 6C–H**). Compared to H/R group, the mRNA and protein expression of ATF4, HIF1α, and VEGF in H/R+Lir group was significantly increased. Compared to H/R group, the mRNA and protein expression levels of ATF4, HIF1α, and VEGF in H/R+Lir group were significantly increased (P < 0.01; **Figure 6C–H**). However, the expression of ATF4, HIF1α, and VEGF mRNA and protein was significantly decreased after the application of GSK2606414 (P < 0.01; **Figure 6C–H**). The mRNA and protein expression levels of ATF4, HIF1α, and VEGF in H/R+G+Lir group were not significantly different from H/R+G group. Compared to H/R+Lir group, the mRNA and protein expression levels in the H/R+G+Lir group were significantly decreased (P < 0.01; **Figure 6C–H**). The above results indicated that the CNPY2-PERK pathway is involved in the angiogenesis of Lir on HUVECs exposed to H/R.

## Discussion

MI is a prominent cardiovascular disease with high incidence and low survival rate (Boateng and Sanborn, 2013). At present, although the medications and surgical treatments have limited damage to the heart and improved clinical symptoms of patients, there are still considerable technical limitations and complications preventing full recovery after MI. As the cause of MI is a blockage of blood flow to the heart, an increase of functional coronary artery branch or collateral circulation through angiogenesis would help restore myocardial blood supply and improve myocardial function (Lutter et al., 2004). An *in vitro* culture of vascular endothelial cells is a good experimental model for studying angiogenesis. In this study, we found that the CNPY2-PERK pathway is involved in HUVEC angiogenesis after H/R. Lir treatment further promoted angiogenesis through this pathway. These findings provided a theoretical basis for the use of Lir after MI.

Cardiac ischemia and hypoxia injury after MI is a serious health burden, and its mechanism has not been fully understood. At present, its pathogenesis involves ERS response, mitochondrial damage, ion balance disorder, oxygen free radical activation, endothelial cell injury, and apoptosis and antiapoptosis imbalance (Heusch, 2015; Santos-Gallego et al., 2016). Signaling pathways are intertwined and mutually promoted, forming a pathogenic link of myocardial dysfunction. Inhibition of one or more pathogenic links may be a breakthrough in improving the prognosis of MI. In particular, collateral vascular dysfunction is an important part of myocardial necrosis in the infarcted area (Thiagarajan et al., 2017). Vascular growth is in a relatively balanced state under normal conditions (Morbidelli et al., 2018). Under ischemia and hypoxia, the ability of vascular endothelial cells to divide, proliferate, and migrate is improved and angiogenesis is promoted (Thiagarajan et al., 2017). Compensatory angiogenesis to establish good collateral circulation and bridging of blood vessels in ischemic area is of great importance for the survival of the myocardium.

Angiogenesis is a complex and closely regulated process. Currently, growth factors with potential angiogenic effects mainly include VEGF, fibroblast growth factor (FGF), platelet-derived growth factor, and so on (Thiagarajan et al., 2017). Among them, VEGF is the most potent growth factor that promotes vascular endothelial cell growth (Crafts et al., 2015). VEGF specifically and strongly promotes endothelial cell expansion, proliferation, and migration, which is directly related to angiogenesis (Petyunina et al., 2018). Therefore, VEGF expression also affects angiogenesis. HIF1α is another key regulator of cardiac angiogenesis during ischemia (Pugh and Ratcliffe, 2003; Kido et al., 2005; Shohet and Garcia, 2007; Chen and Sang, 2016). VEGF is one of the target genes regulated by HIF1α under hypoxic-ischemic conditions (Zimna and Kurpisz, 2015). HIF1α promotes endothelial cell proliferation and migration by promoting the expression of VEGF and improves cardiac function (Cheng et al., 2016). The results of this study showed that after H/R stimulation the activity of HUVECs was significantly increased, and the secretion of HIF1α and VEGF was also significantly increased. All these results suggested that H/R could promote the angiogenesis of HUVECs. Enhanced expression of HIF1α and VEGF was further confirmed at the mRNA and protein levels, suggesting that H/R indeed promotes the production of angiogenic factors and activates the angiogenic ability of HUVECs. These experiments confirmed that H/R enhanced angiogenesis.

GLP-1 is an intestinal insulin-stimulating peptide with functions such as reducing blood sugar and improving insulin resistance (Ahren, 2019). GLP-1 also slows gastric emptying, reduces appetite by acting directly on the brain, reduces blood pressure, modifies cardiac metabolism, and improves glucose-stimulated insulin secretion (Drucker, 2018). The study found that GLP-1 mimetics, including Lir (Chen et al., 2016) and exenatide (Lonborg et al., 2012), ameliorate myocardial H/R injury and reduce MI size. In particular, Lir has received increasing attention in recent years for its cardioprotective effect in addition to the traditional glucose-lowering ability. Lir was found to inhibit myocardial apoptosis and reduce infarct size in a dose-dependent manner in rats after MI (Chen et al., 2016; Hu et al., 2017). Lir improved cardiac function after MI by activating cAMP in mice (Huang et al., 2018). Lir may also play a protective role in MIRI by regulating intracellular calcium (Hu et al., 2017). Lir promotes angiogenesis in palmitate-stressed HUVECs (Ke et al., 2016). The application of Lir may promote angiogenesis to improve cardiac function (Qi et al., 2017). GLP-1 agonist is a promising drug for cardiac protection (Giblett et al., 2016). In this study, we found that 100 nM Lir had no significant effect on normal HUVECs in 24 h. After H/R stimulation, Lir significantly increased the expression of HIF1α and VEGF in HUVECs stimulation and promoted angiogenesis.

GLP-1 receptor (GLP-1R) is widely distributed in the body, but its existence in myocardial tissue has been controversial. Recent studies have shown that GLP-1R is restricted to atrial myocytes and not expressed in ventricular myocytes (Pyke et al., 2014; Richards et al., 2014). To date, it has been controversial whether Lir has direct myocardial protection. Therefore, we need to reevaluate the role of GLP-1R in myocardial protection. Recent studies have found that Lir has potential cardiovascular protection through both GLP-1R-dependent and -independent mechanisms (Giblett et al., 2016). Recent literature has found that UPR is closely related to angiogenesis (Urra and Hetz, 2014; Binet and Sapieha, 2015). Intracellular calcium overload and vascular endothelial cell dysfunction are important pathogenesis of MI (Groenendyk et al., 2013; Zhang et al., 2017). Maintaining intracellular calcium homeostasis and improving vascular endothelial dysfunction can effectively prevent further myocardial injury. The ER is the site of protein synthesis and modification as well as maintenance of calcium homeostasis (Wang et al., 2016). It is also the main site for intracellular protein synthesis, translation, and folding. Tissue ischemia leads to calcium imbalance, ER dysfunction, and the accumulation of misfolded and unfolded proteins in the ER lumen, which activates the highly conserved UPR (Liu et al., 2018a). UPR determines cell survival or death under ERS (Shaheen, 2018). It improves the ability of ER to process and refold proteins, reduces the load of ER, maintains cell homeostasis, and protects cells from prolonged disruption (Binet and Sapieha, 2015). The UPR is mainly mediated by three ER transmembrane proteins: PERK, IRE1, and ATF6 (Lv et al., 2018). In physiological state, these three markers bind to immunoglobulin heavy-chain binding protein GRP78 and are inactive. However, the occurrence of UPR after tissue ischemia and hypoxia triggers the dissociation of three proteins from GRP78, enhances protein folding, and promotes unfolded protein degradation. These efforts eliminate ERS, maintain cell function, and promote cell survival (Luchetti et al., 2017). Recently, a study identified that the IRE1α-XBP-1, PERK-ATF4, and ATF6 pathways constitute a novel upstream regulatory pathway for angiogenesis by regulating VEGF transcription (Ghosh et al., 2010). PERK was the driving factor of VEGF and FGF2 coding after ischemic stress (Philippe et al., 2016). VEGF promoted endothelial cell survival and angiogenesis through ATF6 and PERK signaling (Karali et al., 2014). These results established an important role for UPR in angiogenesis. Our experimental results showed that the expression of UPR marker protein GRP78 in HUVECs increased significantly after H/R stimulation, suggesting the occurrence of UPR. Subsequently, we detected the expression of UPR transmembrane proteins PERK and ATF4. Experimental results showed that the expression of p-PERK and ATF4 in HUVECs was significantly increased after H/R stimulation. The application of Lir significantly increased the expression levels of p-PERK and ATF4 after H/R stimulation. The experimental results suggested that Lir might promote angiogenesis through the UPR pathway.

However, it is unclear how to activate UPR to promote angiogenesis. Recent studies have found that CNPY2 played a key role in ERS induced by liver injury (Hong et al., 2017). CNPY2 located on the ER membrane was identified as a novel regulator of PERK. CNPY2 is released from GRP78 after ERS and activates the PERK-CHOP pathway of UPR (Hong et al., 2017). In vitro studies have shown that CNPY2 enhanced neurite outgrowth in neurons (Bornhauser et al., 2003). CNPY2 is also highly expressed in cardiomyocytes (Hatta et al., 2014). CNPY2 inhibits the transition of hypertrophic cardiomyopathy from compensatory hypertrophy to dilated heart failure (Guo et al., 2015a). CNPY2 enhanced angiogenesis and promoted smooth muscle cell migration and proliferation (Guo et al., 2015b). In addition, CNPY2 is also a secreted angiogeneic growth factor regulated by HIF1α, which promotes the migration and proliferation of smooth muscle cells (Guo et al., 2015b). Interestingly, CNPY2 maintains HIF1α expression in the injured heart, thereby potentially preserving angiogenesis. This study found that in HUVECs H/R significantly increased CNPY2 expression and secretion, which was further enhanced by the application of Lir. These results confirmed that CNPY2 played an important role after H/R and implied that it may also be involved in the up-regulation of PERK in HUVECs.

The application of Lir significantly increased CNPY2 in HUVECs after H/R and increased the expression levels of UPR pathway-related proteins: GRP78, p-PERK, and ATF4. At the same time, Lir increased the expression of HIF1α and VEGF in HUVECs, which reflected enhanced angiogenesis. These suggested that Lir might increase cell angiogenic potential after H/R stimulation through the CNPY2-PERK pathway. To further confirm the experimental results, we added a PERK inhibitor GSK2606414. It was found that PERK inhibitor significantly reduced the mRNA and protein expression of ATF4, HIF1α, and VEGF in HUVECs after H/R. However, Lir did not significantly increase the expression of ATF4, HIF1α, and VEGF mRNA and protein using the PERK inhibitor. The above experiments confirmed that the effect of Lir in promoting endothelial cell angiogenesis likely acts through the CNPY2-PERK pathway after H/R injury.

In conclusion, our study demonstrates that the CNPY2-PERK pathway is involved in the mechanism of VEGF expression after H/R injury in HUVECs. Lir increased the expression of VEGF in HUVECs through this pathway, which promoted endothelial cell angiogenesis and protected HUVEC from H/R damage. However, it is possible that other possible mechanisms connecting GLP-1R and UPR exist and have not been fully elucidated by our study. Similarly, the effect of Lir after H/R injury was only tested at 100 nM for 24 h. Its effect in endothelial cells beyond 24 h is unknown. It is likely that Lir can promote angiogenesis through other branches of UPR or additional mechanism downstream of GLP-1R. Further study may look into the proposed mechanisms in elucidating the effect of Lir on angiogenesis. Nonetheless, our results provide a new theoretical basis for the use of Lir in the treatment of MI.

## Data Availability

The raw data supporting the conclusions of this manuscript will be made available by the authors, without undue reservation, to any qualified researcher.

## Author Contributions

FT, HL, and CL conceived and designed the experiments. HL, YL, and RM supervised the project. CL, YD, JH and NS performed the study and collected data. JW, TC, JH and NS analyzed the data. CL and YL wrote the paper. YL, XL, and XG drafted the manuscript. TF and HL revised the manuscript. All authors gave the final approval and agreed to be accountable for all aspects of the work.

## Funding

This work was supported by the High-Priority Health Projects of Tianjin (16KG146), Tianjin Major Science and Technology Projects (17ZXMFSY00200), China Youth Clinical Research Fund-VG Fund (2017-CCA-VG-021), and 2018 Tianjin Medical Association Anesthesiology Branch Youth Research and Development Fund Project.

## Conflict of Interest Statement

The authors declare that the research was conducted in the absence of any commercial or financial relationships that could be construed as a potential conflict of interest.
